# Comparative changes in treatment practices and clinical outcomes following implementation of a prospective payment system: the STEPPS study

**DOI:** 10.1186/s12882-015-0059-8

**Published:** 2015-05-01

**Authors:** Keri L Monda, Parveen Nedra Joseph, Peter J Neumann, Brian D Bradbury, Robert J Rubin

**Affiliations:** Center for Observational Research, Amgen, Inc, One Amgen Center Drive MS 24-2-A, Thousand Oaks, CA 91320-1799 USA; Center for the Evaluation of Value and Risk in Health, Institute for Clinical Research and Health Policy Studies, Tufts Medical Center, Boston, MA USA; Division of Nephrology and Hypertension, Georgetown University School of Medicine, Washington, DC USA

**Keywords:** Anemia management, Clinical care indicators, Dialysis, Prospective Payment System, Reimbursement, Small dialysis organization

## Abstract

**Background:**

The aim of the US dialysis Prospective Payment System bundle, launched in January 2011, was reduction and more accurate prediction of costs of services, whilst maintaining or improving patient care. Dialysis facilities could either adopt the bundle completely (100%) in the first year of launch, or phase-in (25%) over four years. Differences in practice patterns and patient outcomes were hypothesized to occur in facilities that phased-in 25% compared to those that did not.

**Methods:**

Data are from STEPPS, a study of 51 small dialysis organization facilities designed to describe trends in dialytic treatment before and after bundle implementation. Baseline was defined as October-December 2010; follow-up as January-December 2011. Facility- and patient-level data were collected at enrollment and regularly thereafter. Cox proportional hazards and linear multi-level models were used to estimate the effect of opting-in 25% (vs. 100%) on practice patterns and clinical outcomes.

**Results:**

12 facilities (patient n = 346) opted-in 25% and 37 facilities (patient n = 1296) opted-in 100% to the dialysis bundle. At baseline, patients at 25% facilities were primarily covered by Medicare, were more likely to be black, and were receiving higher monthly epoetin alfa (EPO) doses. Throughout 2011, patients in 100% facilities received lower monthly EPO doses, and had lower mean hemoglobin concentrations; hospitalization and mortality rates were numerically lower in 25% facilities but not statistically different.

**Conclusions:**

The economic pressure for dialysis providers to work within an expanded composite rate bundle whilst maintaining patient care may be a driver of practice indicator outcomes. Additional investigations are warranted to more precisely estimate clinical outcomes in patients attending facilities enrolling into the bundle 100% relative to the previous fee-for-service framework.

**Electronic supplementary material:**

The online version of this article (doi:10.1186/s12882-015-0059-8) contains supplementary material, which is available to authorized users.

## Background

In the United States, over 400,000 individuals have end stage renal disease (ESRD) requiring dialysis treatment to sustain life [1]. Patients suffer from high co-morbidity burden, are frequently hospitalized, and face a mortality rate of 194 per 1000 patient-years [1]. Given their health status, frequent treatment schedule, and the growth of the population [1,2], costs of treating ESRD patients, estimated at $34.3 billion in 2011, have placed a substantial budgetary burden on the Centers for Medicare & Medicaid Services (CMS) [1].

In January 2011 the updated prospective payment system (PPS) for ESRD (i.e. the “expanded dialysis composite rate bundle”) was launched, with the objective of reducing costs of services and more accurately predicting expenditures while maintaining or improving patient care [3]. Injectables such as epoetin alfa (EPO), darbepoetin alfa (DPO), iron, and activated vitamin D analogs which had previously been reimbursed independently were now included in the bundled payment. Dialysis facilities were given the option to enroll immediately (100%) into the dialysis bundle in 2011, or to phase-in gradually (25% per year) over four years. Most facilities (92.2%) enrolled 100%; large dialysis organizations (LDOs) enrolled over 99% of their facilities immediately, but small (SDOs) and independent dialysis organizations (IDOs), and hospital-based facilities opted-in to a lesser extent [1]. This choice presented the opportunity to investigate the effect of different economic drivers on practice patterns and patient outcomes, as has been suggested [4-6], in facilities choosing to phase-into the dialysis bundle versus those opting-in fully. Results might be particularly informative as CMS seeks to bundle payments for a number of medical conditions.

Because LDOs have both a larger patient-base over which to distribute costs and are able to negotiate volume rebates, it was postulated that they may have an advantage over the IDOs and SDOs in managing the financial implications of the PPS [6-8]. Shifts in practice patterns and clinical indicators may be more evident in smaller organizations, particularly in the first year of the transition.

We used data from the Study to Evaluate the Prospective Payment System Impact on Small Dialysis Organizations (STEPPS), a study designed to describe trends in dialytic treatment before and after implementation of the expanded bundle in a representative sample of SDO facilities.

## Methods

### Study design

STEPPS was a multi-center prospective observational cohort study of patients receiving care in 51 dialysis facilities that were members of SDOs within the United States. An SDO was defined as a dialysis provider that was either a stand-alone facility or part of a chain with 50 or fewer facilities. Facility and patient selection for STEPPS has been described previously [9]. Briefly, facilities were selected from all free-standing US dialysis facilities circa 2006 that qualified as SDOs. Patients were at least 18 years old, receiving dialysis, and had provided informed consent. During the study, patients who discontinued were replaced with patients within the same facility. The study received institutional review board approval from all participating facilities.

Data were collected between October 2010 and September 2012. For this analysis, we used data between October 2010 and December 2011. The baseline period was defined as October 1, 2010 to December 31, 2010; follow-up was from January 1, 2011 to December 31, 2011 (to capture the first year of the expanded PPS when facilities phasing-in were enrolled 25%).

### Data collection

The outcomes of interest for this study include ESA dosing patterns (type/route), medication use, hemoglobin concentrations and other laboratory parameters, hospitalization events, and mortality. Patient- and facility-level data were obtained using standardized electronic data collection forms. Patient-level demographic, comorbidity, anthropometric, laboratory, and medication data were collected at enrollment and scheduled intervals thereafter. Data on hospitalization events (dates and diagnoses), death, and other censoring events were recorded by the facility throughout follow-up. Although data on red blood cell transfusion were collected, we know these data to be under-reported [9], and that this under-reporting differs by opt-in status (25% facilities were three times more likely to report zero transfusions than 100% facilities). Facility-level characteristics were collected at enrollment and quarterly thereafter. Data validation included checks for implausible values and verification of source documentation (e.g. hospital records) from a sample of patients at each facility.

### Data considerations

Baseline characteristics are described for patients on study between October and December 2010. Study participants were required to be receiving in-center hemodialysis (HD); those on peritoneal dialysis or home hemodialysis were excluded due to small numbers. Dialysis modality was assessed monthly. For patients switching modality, only patient-time on HD was included. For the 3% of patient-months with missing modality information, 17% of this missing data were imputed based on information available in the prior and subsequent months.

Erythropoiesis stimulating agent (ESA) use is reported as the total monthly dose administered in the last eligible month of each quarter. For this analysis, only epoetin alfa values are reported as no darbepoetin alfa use was recorded in the 25% facilities. Laboratory values are reported as the last non-missing value from the last eligible month in each quarter; values outside an *a priori* specified acceptable range were set to missing. Medications such as iron, vitamin D, cinacalcet, and phosphate binders are reported as any use (yes/no) within each quarter. Dosing route was obtained for ESAs [intravenous (IV) vs. subcutaneous (SC)] and for iron and vitamin D (IV vs. oral); both are reported as last administered route within the quarter.

### Statistical analysis

For each patient, person-time accumulated from January 1, 2011 or date of eligibility to the end of follow-up defined as the first of the following events: death, withdrawal from study, facility transfer, or December 31, 2011. Event rates were estimated as first events (mortality, mortality/hospitalization composite) or total events (all hospitalizations) divided by the person-time at risk. For patients who switched dialysis modality during follow-up, person-time continued to accumulate for 60 days post-switch to avoid under-assessment of events unrelated to modality switch. Person-time was not extended past December 31, 2011.

Descriptive statistics are presented as counts and proportions for categorical variables, and means and standard deviations for continuous variables. We assessed imbalance in baseline patient- and facility-level characteristics between the 25% and 100% facilities in two ways: via p-value associated with *t*-test, chi-square, or Wilcoxon, as appropriate, and by calculating the standardized difference which is comparable to the z-score of a standard normal distribution and, unlike the p-value, is unaffected by sample size [10]; a difference of > |0.20| was considered meaningful. Associations with time (quarterly trends) over 2011 as well as Q4 2010 to Q4 2011 comparisons were modeled using generalized estimating equations with an unstructured covariance matrix accounting for clustering within both facilities and patients over time.

The effect of receiving care at a 25% (vs. 100%) facility was estimated using Cox proportional hazards regression for mortality and a mortality/hospitalization composite outcome (hereinafter referred to as “composite”); using negative binomial regression for hospitalization rate; and using linear mixed effects regression for monthly hemoglobin concentration and EPO dose. Results from three levels of adjustment are presented: unadjusted, parsimonious, and extended. Parsimonious models were built by including all time-invariant variables with a baseline standardized difference of > |0.20| and then performing backwards selection until only those that produced a change of >10% in the effect estimate when removed from the model remained [11]. We elected to not evaluate time-dependent variables that showed imbalance across 25% and 100% facilities (limited to vitamin D use, ferritin, transferrin saturation [TSAT], and parathyroid hormone [PTH], see Table 1) as potential confounders because i) many of these factors may be on the causal pathway between the medical interventions and the clinical outcomes of interest and, ii) there were a number of patients eligible during the follow-up period without baseline data (253 replacement patients and nine from the facility that enrolled in January 2011) who would have been excluded from all models, thus impacting the precision of our estimates. Extended models added in the patient-level variables age, sex, race, and time on dialysis, and the facility-level characteristic percent with Medicare as primary payer. Outcomes were also stratified by race. Baseline values for covariates were used in all adjusted models. All statistical models accounted for clustering within facilities and within individuals over time, as appropriate. All analyses were conducted using SAS V9.2 (Cary, NC).

**Table 1:** **Baseline facility- and patient-level characteristics in the STEPPS study by dialysis facility 25% and 100% opt-in status**

**BASELINE CHARACTERISTICS N (%); mean ± SD**	**25% (n = 346, 12 facilities)**	**100% (n = 1296, 37 facilities)**	**p-value**	**|Standardized Difference|**
**Facility-level characteristics**				
South (vs.non-South) geographic region	5 (41.7)	17 (45.9)	0.80	0.09
Urban (vs.rural)	10 (83.3)	28 (75.7)	0.71	0.19
For profit status	12 (100.0)	30 (81.1)	0.17	0.68
Percent Medicare primary payer	78.1 ± 14.9	64.0 ± 17.6	0.02	0.86^d^
**Patient-level characteristics**				
Age (years)	60.6 ± 15.6	61.6 ± 14.9	0.29	0.06
Male sex	201 (58.1)	738 (56.9)	0.70	0.02
Race				
White	130 (37.6)	743 (57.3)	0.00	0.42^d^
Black	137 (39.6)	303 (23.4)		
Other/Unknown	79 (22.8)	250 (19.3)		
Primary Cause of CKD				
Diabetes	151 (43.6)	604 (46.6)	0.05	0.15
Hypertension	110 (31.8)	328 (25.3)		
Other/unknown	85 (24.6)	364 (28.1)		
BMI (kg/m^2^)	28.6 ± 6.7	29.2 ± 7.7	0.16	0.08
Years on dialysis	4.1 ± 3.7	3.1 ± 3.1	<0.0001	0.29^d^
Medical History				
Congestive Heart Failure	111 (32.1)	359 (27.7)	0.11	0.10
Hypertension	288 (83.2)	1121 (86.5)	0.12	0.09
Diabetes	148 (42.8)	623 (48.1)	0.08	0.11
Cancer (other than skin)	31 (9.0)	157 (12.1)	0.10	0.10
Vascular access type^a^				
Arteriovenous fistula/graft	287 (82.9)	1013 (78.3)	0.06	0.12
Venous catheter	59 (17.1)	281 (21.7)		
**Laboratory values** ^a^				
Hemoglobin (g/dL)	11.3 ± 1.3	11.2 ± 1.3	0.26	0.07
Hemoglobin group				
<10 g/dL	41 (11.8)	194 (15.0)	0.15	0.12
10 – 12 g/dL	204 (59.0)	784 (60.5)		
≥12 g/dL	99 (28.6)	317 (24.5)		
PTH (pg/dL)	443.0 ± 344.3	358.4 ± 306.8	<0.0001	0.26^e^
Calcium (mg/dL)	8.9 ± 0.8	9.0 ± 0.7	0.02	0.14
Phosphorus (mg/dL)	5.3 ± 1.6	5.3 ± 1.6	0.68	0.03
TSAT (%)	28.7 ± 11.0	31.4 ± 12.3	<0.0001	0.23^e^
Ferritin (ng/mL)	642.6 ± 388.2	754.7 ± 466.8	<0.0001	0.26^e^
Albumin (g/dL)	3.8 ± 0.4	3.8 ± 0.4	0.44	0.05
**ESA administration** ^a,b^				
EPO monthly dose (units)	62471 ± 61748	53655 ± 55605	0.02	0.15
IV (vs. SC) ESA route	299 (92.6)	1092 (92.0)	0.74	0.02
**Other medications** ^c^				
Iron use				
IV only	239 (69.1)	891 (68.8)	0.69	0.08
Oral only	1 (0.3)	11 (0.8)		
Both IV and Oral	5 (1.4)	17 (1.3)		
No iron use	101 (29.2)	377 (29.1)		
Vitamin D use				
IV only	284 (82.1)	949 (73.2)	<0.0001	0.32^e^
Oral only	0 (0.0)	37 (2.9)		
Both IV and Oral	9 (2.6)	78 (6.0)		
No Vitamin D use	53 (15.3)	232 (17.9)		
Cinacalcet use	82 (23.7)	294 (22.7)	0.69	0.02
Phosphate binder use	232 (67.1)	791 (61.0)	0.04	0.13

^a^Last non-missing value in the baseline period; ^b^Among ESA users; ^c^Within the baseline period; ^d^Time-invariant variable evaluated as potential confounder in parsimonious modeling; ^e^Time-variant variable not evaluated as potential confounder in parsimonious modeling.

BMI, body mass index; TSAT, transferrin saturation; PTH, parathyroid hormone; EPO, epoetin alfa; IV, intravenous; SC, subcutaneous.

## Results

STEPPS includes 51 dialysis facilities. At baseline, 12 facilities (patient n = 346) opted-in 25% and 37 facilities (patient n = 1296) opted-in 100% to the dialysis bundle. Two facilities are not represented at baseline: one did not include any eligible patients and the other enrolled in January 2011. While facility-level characteristics at baseline are mostly similar between 25% and 100% facilities, 25% facilities included a larger percentage of patients with Medicare as primary payer. Patients receiving treatment at 25% facilities tended to be black, received dialysis longer, and received higher monthly EPO doses (Table 1).

Between the beginning of Q4 2010 and the end of Q1 2011, STEPPS lost one 25% facility due to closure and gained one 100% facility that enrolled in January 2011. Quarterly patient-level characteristics are presented in Table 2. In both 25% and 100% facilities, EPO doses declined, the percentage of patients with hemoglobin concentrations ≥12 g/dL declined while those with hemoglobin <10 g/dL increased, the percentage of patients with ESAs dosed SC (vs. IV) increased, mean ferritin levels increased, and phosphate binder use increased. In all cases, changes were more pronounced in 100% versus 25% facilities. Additionally in 100% facilities, cinacalcet use increased, and patients were more likely to be treated with oral forms of vitamin D and iron.

**Table 2:** **Trends by quarter within 2011 in medication use and laboratory values by dialysis facility 25% and 100% opt-in status**

	**25% facilities (N = 11)**					**100% facilities (N = 38)**				
**N (%), Mean ± SD**	**Q1 2011 (n = 346)**	**Q2 2011 (n = 325)**	**Q3 2011 (n = 311)**	**Q4 2011 (n = 298)**	**p-trend**	**Q1 2011 (n = 1266)**	**Q2 2011 (n = 1236)**	**Q3 2011 (n = 1166)**	**Q4 2011 (n = 1125)**	**p-trend**
**ESA administration** ^a^										
EPO monthly dose (units)	67624 ± 64409	62226 ± 64937	59134 ± 64567	54814 ± 57216	<0.0001	46294 ± 48614	46071 ± 47388	37973 ± 41916	40892 ± 43618	<0.0001
IV EPO monthly dose (units)	68687 ± 64337	63694 ± 65008	58813 ± 64117	55029 ± 56670	<0.0001	45455 ± 47892	45503 ± 46503	37300 ± 41087	42225 ± 45218	<0.0001
SC EPO monthly dose (units)	54904 ± 65232	46254 ± 63186	62404 ± 70244	52500 ± 64087	0.89	53561 ± 54168	51289 ± 54835	43056 ± 47596	33294 ± 32131	0.59
SC (vs.IV) ESA route^b^	25 (7.7)	26 (8.4)	26 (8.9)	24 (8.5)	0.22	112 (9.6)	105 (9.1)	122 (11.4)	152 (14.8)	<0.0001
**Other medications** ^**c**^										
Cinacalcet use	97 (28.0)	92 (28.3)	85 (27.3)	81 (27.2)	0.43	336 (26.5)	370 (29.9)	359 (30.8)	356 (31.6)	<0.0001
Phosphate binder use	237 (68.5)	226 (69.5)	229 (73.6)	214 (71.8)	0.85	849 (67.1)	883 (71.4)	872 (74.8)	827 (73.5)	<0.0001
Iron use	251 (72.5)	221 (68.0)	216 (69.5)	223 (74.8)	0.9	903 (71.3)	917 (74.2)	817 (70.1)	752 (66.8)	0.004
Oral (vs.IV) iron route^b^	1 (0.4)	1 (0.5)	2 (0.9)	3 (1.3)	-	8 (0.9)	13 (1.4)	14 (1.7)	13 (1.7)	0.21
Vitamin D use	294 (85.0)	275 (84.6)	254 (81.7)	246 (82.6)	0.20	975 (77.0)	967 (78.2)	927 (79.5)	914 (81.2)	0.001
Oral (vs.IV) vitamin D route^b^	11 (3.7)	6 (2.1)	3 (0.2)	4 (2.1)	0.03	151 (15.5)	202 (20.9)	219 (23.6)	226 (24.7)	<0.0001
**Laboratory values** ^a^										
Hemoglobin (g/dL)	11.3 ± 1.2	11.3 ± 1.3	11.3 ± 1.3	11.0 ± 1.1	0.0002	11.1 ± 1.3	11.0 ± 1.2	10.9 ± 1.3	10.7 ± 1.2	<0.0001
Hemoglobin <10 g/dL	41 (11.8)	37 (11.4)	41 (13.2)	40 (13.4)	0.34	229 (18.1)	226 (18.3)	251 (21.5)	268 (23.8)	<0.0001
Hemoglobin ≥12 g/dL	96 (27.7)	92 (28.3)	87 (28.0)	52 (17.4)	0.004	314 (24.8)	225 (18.2)	210 (18.0)	132 (11.7)	<0.0001
PTH (pg/dL)	393.4 ± 332.9	500.8 ± 404.4	412.1 ± 330.7	458.7 ± 355.3	0.70	380.2 ± 305.9	399.2 ± 315.4	414.3 ± 321.8	436.3 ± 343.7	<0.0001
Calcium (mg/dL)	8.8 ± 0.8	8.8 ± 0.8	8.9 ± 0.7	8.9 ± 0.7	0.06	8.9 ± 0.6	8.9 ± 0.7	8.9 ± 0.7	8.8 ± 0.7	<0.0001
Phosphorus (mg/dL)	5.2 ± 1.5	5.4 ± 1.7	5.4 ± 1.6	5.2 ± 1.5	0.63	5.2 ± 1.6	5.3 ± 1.6	5.3 ± 1.7	5.2 ± 1.6	0.14
TSAT (%)	30.8 ± 12.9	30.9 ± 12.7	33.0 ± 13.8	30.1 ± 11.4	0.82	32.7 ± 12.5	32.9 ± 12.6	33.5 ± 12.3	31.6 ± 11.7	0.19
Ferritin (ng/mL)	677.7 ± 383.2	663.6 ± 409.8	739.6 ± 405.9	693.8 ± 364.0	0.001	826.8 ± 438.0	849.2 ± 456.3	933.5 ± 471.8	922.6 ± 472.8	<0.0001

^a^Assessed during last eligible month in each quarter; ^b^Last administered route in each quarter among users; ^c^Any use during the quarter.

EPO, epoetin alfa; IV, intravenous; SC, subcutaneous; ESA, erythropoiesis stimulating agent; TSAT, transferrin saturation; PTH, parathyroid hormone.

**Figure 1:**
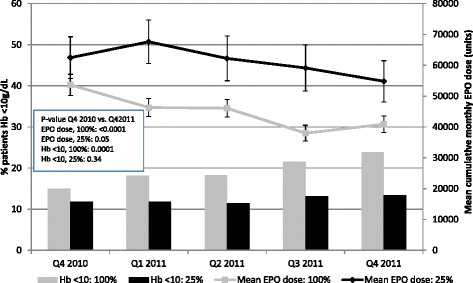
Unadjusted quarterly mean (95% confidence interval) epoetin alfa dose (lines) and percentage of subjects with hemoglobin concentrations <10 g/dL (bars) by 25% and 100% dialysis facility opt-in status. Hb, hemoglobin; EPO, epoetin alfa.

Figure 1 presents the quarterly mean monthly EPO dose and percentage of patients with hemoglobin concentrations <10 g/dL between baseline and Q4 2011. By Q4 2011, 24% of patients at 100% facilities had hemoglobin concentrations <10 g/dL (Q4 2010 vs. Q4 2011, p = 0.0001) compared to 13% of patients at 25% facilities (p = 0.34). As compared to patients in 25% facilities, patients in 100% facilities were receiving lower EPO doses at baseline and show a greater absolute and relative decline in monthly dose (Q4 2010 vs. Q4 2011, p < 0.0001 and p = 0.05 in 100% and 25% facilities, respectively).

**Figure 2:**
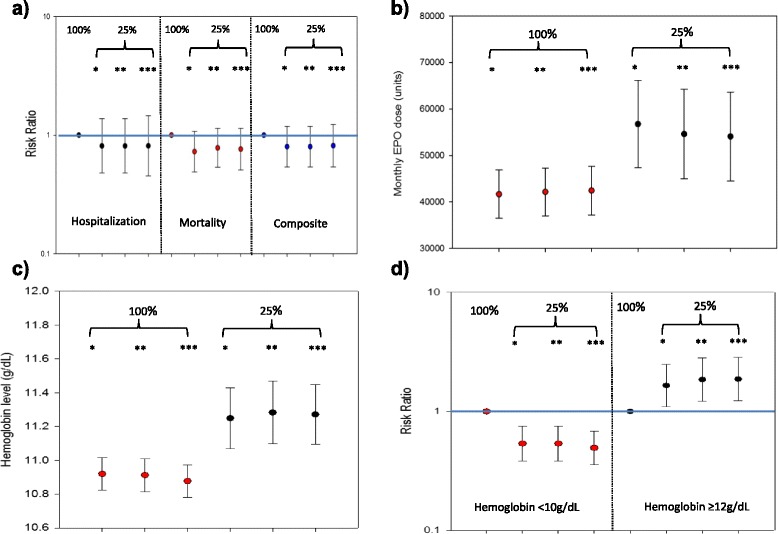
The effect of dialysis facility opt-in status on outcomes. **A**. Hospitalization, mortality, and their composite. **B**. Monthly EPO dose. **C**. Hb concentration. **D**. Percentage of patients with Hb <10 and >=12 g/dL. *Unadjusted; **Adjusted for race (mortality), insurance (EPO dose, Hb outcomes); ***Adjusted for insurance, race, age, sex, and time on dialysis. EPO, epoetin alfa; Hb, hemoglobin. Bars represent 95% confidence intervals.

We assessed rates of mortality, hospitalization, and a mortality/hospitalization composite in 25% and 100% facilities over 2011. Unadjusted annual rates for mortality and hospitalization were higher in 100% facilities, compared to 25% facilities [rate (95% CI) per 100 person-years for mortality: 14.0 (11.9, 16.2) and 10.2 (6.7, 13.8); hospitalization: 112.7 (106.6, 118.9) and 92.3 (81.6, 103.0); composite: 75.1 (69.4, 80.9) and 60.3 (50.5, 70.2), respectively]. Causes of death were well-balanced across facility type, with cardiovascular as primary (41.4% and 40.3% in 25% and 100% facilities, respectively) followed by unknown cause (34.5% and 33.6% in 25% and 100% facilities, respectively). Similarly, causes of hospitalization were relatively well-balanced across facility type, although cardiovascular-related reasons were nominally higher in 100% facilities (24.1% vs. 13.6%, results not shown). After adjustment, patients receiving treatment in facilities that opted-in 25% (vs. 100%) experienced numerically fewer deaths [RR (95% CI) = 0.78 (0.54, 1.14)], fewer hospitalizations [RR (95% CI) = 0.81 (0.48, 1.37)], and fewer composite events [RR (95% CI) = 0.80 (0.54, 1.19)], received higher mean monthly EPO doses [54,610 (44,963, 64,256) vs. 42,156 (37,005, 47,307) units], achieved higher mean hemoglobin concentrations [11.3 (11.1, 11.5) vs. 10.9 (10.8, 11.0) g/dL], had a smaller percentage of patients with hemoglobin concentrations <10 g/dL [RR (95% CI) = 0.54 (0.38, 0.75)] and a greater percentage of patients with hemoglobin concentrations ≥12 g/dL [RR (95% CI) = 1.85 (1.22, 2.83)] (Figure 2a-d).

Mean monthly EPO doses, hemoglobin levels, and the percentage of patients with hemoglobin concentrations <10 g/dL and ≥12 g/dL, stratified by race, are presented in Additional file 1: Figure S1. We found the effect of phasing into the PPS 25% (vs. 100%) on monthly EPO dose was greater among black patients (Additional file 1: Figure S1a). Differences in hemoglobin outcomes in 25% opt-in (vs. 100%) facilities were observed for both white and black patients, but not among patients reporting other race (Additional file 1: Figure S1b-d). As a sensitivity analysis we ran all models limited to the baseline-eligible population in order to evaluate the effect of excluding time-variant parameters with potential imbalance across 25% and 100% facilities and found results to be relatively unchanged, although statistically less precise (results not shown).

## Discussion

STEPPS was a prospective observational cohort study designed to evaluate changes in clinical management and outcomes in patients receiving dialysis within SDOs over the PPS implementation period. We used a contemporaneous comparator group to explore the clinical implications of major changes in reimbursement among two types of facilities: those opting for 100% reimbursement of Medicare patients under the bundled payment system (i.e. experimental group) vs. those electing to receive reimbursement under the bundled payment system in 25% increments over four years (i.e. control group). Leveraging this “natural experiment,” we evaluated the effect of policy changes on dialysis practice patterns and patient outcomes in a representative sample of SDOs during the first year of the expanded composite rate dialysis bundle (the year with the largest differences in financial incentives).

The majority of STEPPS facilities opted fully into the dialysis bundle, with 24% choosing to phase-in 25% over four years. As was anticipated by clinicians, dialysis organizations, and policy analysts [12-15], we observed changes in the use of injectable and oral medications and increases in subcutaneous administration in all facilities consistent with financial incentives; however, these changes were less pronounced in 25% facilities. Similarly, we observed less pronounced changes in laboratory values (hemoglobin levels, serum ferritin and PTH) in 25% facilities. However, because iron and vitamin D doses were not collected, analyses of these data cannot address the comparative effectiveness of IV vs. oral therapies.

While the majority of patient and facility characteristics were relatively balanced among the 25% and 100% facilities before bundle implementation, the few exceptions are revealing. Notably, 25% facilities were more likely to have greater proportions of patients who were black, who were receiving higher monthly EPO doses, and had Medicare as their primary payer. Dialysis providers understand the cost drivers within their facilities; critical evaluation of their patient and payer case-mix and pre-PPS ESA utilization could have easily played a role during the opt-in decision-making process. After statistical adjustment, we continued to observe higher EPO doses and higher mean hemoglobin levels in 25% facilities. However, upon evaluating hospitalizations, mortality, and their composite, we observed effects in the 25% facilities to be numerically lower and directionally consistent, but statistically imprecise. Our findings are consistent with those reported from the United States Renal Data System (USRDS) [1], as well as the DOPPS Practice Monitor (DPM) which was established to evaluate the impact of the dialysis bundle on practice patterns and clinical care [16].

Previously, we found changes in anemia management to be more pronounced among black patients as compared to other racial groups [9]. The DPM has also reported more substantial hemoglobin declines among black patients [17]. While we observe a differential mean monthly EPO dose between those facilities opting-in 25% vs. 100%, adjusting for race attenuates this effect, suggesting that race is confounding the association between opt-in status and dose. While the interplay between race, dose, dose changes, and whether this interplay entered into a facility's decision-making process is complex, the exclusion of race as a case-mix adjuster in the calculation of per-treatment bundled payment has continued to be a source of concern among the renal community given that black patients require higher ESA doses to achieve comparable hemoglobin concentrations [14,18-22].

Outside the US there are has been a limited number of investigations assessing the effects of bundling policies on patient outcomes in the dialysis setting. In 2006, Japan implemented a bundled payment policy to cover outpatient hemodialysis therapy with the stated aim of lowering dialysis payments by 4%. Comparing cross-sections of patients in Japanese DOPPS facilities before and after the implementation of the payment policy, Hasegawa *et al*. report an 11.8% decrease in EPO doses and a concomitant increase in the percentage of patients prescribed IV iron, with no change in mean hemoglobin levels (10.4 g/dL before and after the policy change) [23]. Portugal transitioned in 2008 from a fee-for-service to a capitated payment system for hemodialysis, implemented in conjunction with clinical quality indicators required for reimbursement. Ponce *et al*. evaluated changes in clinical outcomes before and two years after implementation of capitation and observed only minor differences determined to be independent of case-mix [24]. Notably, however, the authors suggested that the bundled payment system incentivized “compliance rather than performance.” In an analysis of the DOPPS data from Germany where dialysis procedure rates were changed in 2002 from per-session to weekly flat rate payments, Kleophas and colleagues report “overall no negative developments” with the reimbursement changes and concomitant implementation of a quality assurance program [25]. In contrast to these investigations that compared practice changes before and after implementation of capitated payment systems, our analysis attempts to compare practice and outcome changes in two contemporary cohorts; one under full transition to a bundled payment system and the other experiencing a staged approach. Consistent with the economic incentives of capitated payments, results suggest more pronounced practice pattern changes in facilities that enrolled in the fully capitated model, with a potentially deleterious effect on clinical outcomes.

Our study is limited in several ways. Sparse data are available prior to PPS implementation during which we evaluated differences in patient- and facility-level characteristics (limited to Q4 2010). Dialysis providers may have begun altering their practice patterns early in 2010, as noted by the USRDS [26] and further evidenced by the rise in home hemodialysis modalities observed [26], and thus our baseline period may not be entirely reflective of the pre-bundle period. However, if practice pattern changes were early and occurring before our ability to capture them, then we would expect our estimates of effect to be biased toward the null. Likewise, there were a number of patients enrolled into STEPPS after the baseline period who are not represented in baseline data. We also excluded the time-variant parameters found to be imbalanced across 25% and 100% facilities as potential confounders in our statistical models, which may bias our estimates if these values affect both the opt-in decision and the outcomes studied. However, similar effect estimates from sensitivity analyses in which these variables were included suggest that confounding was not a major concern. Assessments of hospitalizations and mortality are potentially underreported given that they are reported by the facilities and are not derived from claims or death notification forms. Further, although simple descriptive analyses suggest that patients in 25% facilities are less likely to receive a transfusion than those in 100% facilities (results not shown) we were unable to assess differences in transfusion rates given that data capture differed by facility opt-in status. Next, given the small number of facilities that phased-in 25% and the one year of follow-up time, we have limited power to detect an effect on clinical outcomes; nonetheless, results are directionally consistent despite their lack of precision and provide rationale for further, larger, studies of this nature. Additionally, any study that is of a non-randomized nature is subject to biases that are less controllable. However, patient- and facility-level characteristics were relatively well balanced across facility types, and by comparing at a facility level we gain an increased ability to control for unmeasured factors. Finally, by design, this analysis is limited to the first year of the expanded bundle implementation; future research should assess whether the differential effect diminishes over time by including subsequent years when phasing facilities are subject to increased capitation.

## Conclusions

In summary, STEPPS is composed of a representative sample of SDOs that provides valuable insights into the effects of the ESRD PPS on practice patterns and patient outcomes. CMS’s implementation of the ESRD expanded composite rate dialysis bundle is an important policy change with substantial implications, given the interest in containing health care costs for other disease conditions via introduction of future bundled payment policies. Leveraging the 25% vs. 100% analytic framework allows us to evaluate policy changes on patient care in a timeframe during which dialysis providers were subject to similar pressures but with differential time horizons. Herein, we observe pronounced effects on practice indicators which are thought to be economically driven, and which might have been anticipated, given the dialysis providers’ need to maintain patient care within a capitated payment structure without being at an economic disadvantage as a result of continuing to treat dialysis patients. Nonetheless, we note that the decision to choose whether to opt in 100% or phase-in 25% over four years was likely a complex one, given each center’s unique situation regarding costs, economic acumen and nimbleness (requiring a sophisticated understanding of the QIP and resulting effects on future payment years), patient and payer case-mix, and stated mission. Given that these results are from a relatively small subset of the entire population affected by the dialysis bundle, additional investigations are warranted to provide more precise estimates of the clinical outcomes experienced by patients in centers enrolling into the bundle 100% relative to the previous fee-for-service capitation framework.

## Additional file

Additional file 1: Figure S1. The effect of dialysis facility opt-in status on outcomes, by race. A. Monthly EPO dose. B. Hb concentration. C. Likelihood of having Hb <10 g/dL. D. Likelihood of having Hb >=12 g/dL. *Unadjusted; **Adjusted for insurance (EPO dose, Hb level, percentage of subjects with Hb >=12 g/dL); ***Adjusted for insurance, age, sex, and time on dialysis. EPO, epoetin alfa; Hb, hemoglobin. Bars represent 95% confidence intervals.

## Abbreviations

CMS: Centers for Medicare & Medicaid Services
DPM: DOPPS Practice Monitor
DPO: Darbepoetin alfa
EPO: Epoetin alfa
ESA: Erythropoiesis Stimulating Agents
ESRD: End-stage Renal Disease
HD: Hemodialysis
IDO: Independent Dialysis Organizations
IV: Intravenous
LDO: Large Dialysis Organizations
PPS: Prospective Payment System
PTH: Parathyroid hormone
SC: Subcutaneous
SDO: Small Dialysis Organizations
STEPPS: Study to Evaluate the Prospective Payment System Impact on Small Dialysis Organizations
TSAT: Transferrin saturation
USRDS: United States Renal Data System
