# Supplementary material for: Comparative changes in treatment practices and clinical outcomes following implementation of a prospective payment system: the STEPPS study
Source: BMC Nephrol. 2015 May 1;16:67. doi: 10.1186/s12882-015-0059-8 (PMC4428114; doi:10.1186/s12882-015-0059-8)

Supplementary Figure 1a-d

a)

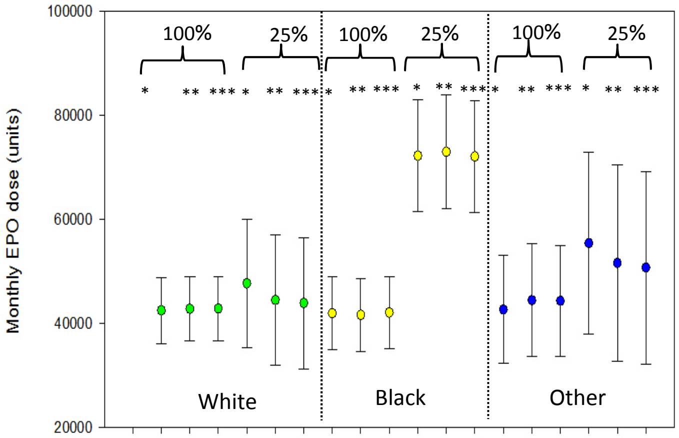

b)

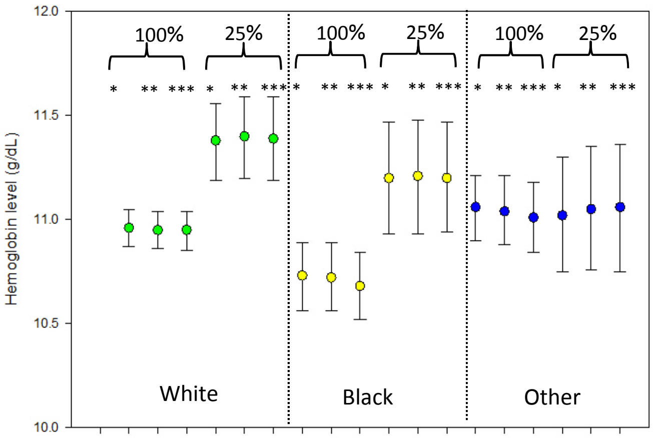

c) Patients with mean hemoglobin <10g/dL

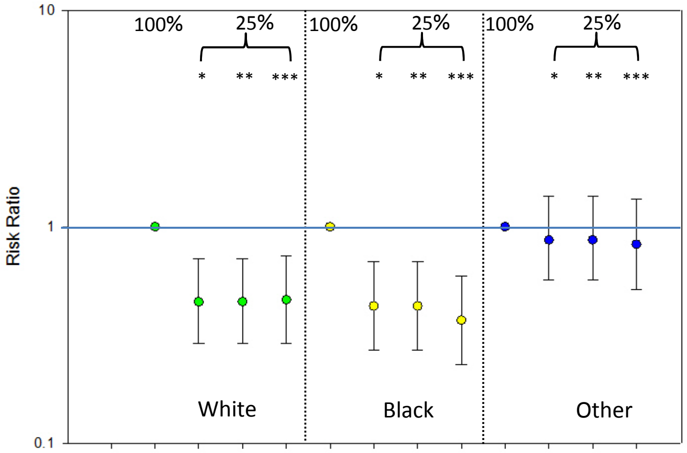

d) Patients with mean hemoglobin ≥12g/dL

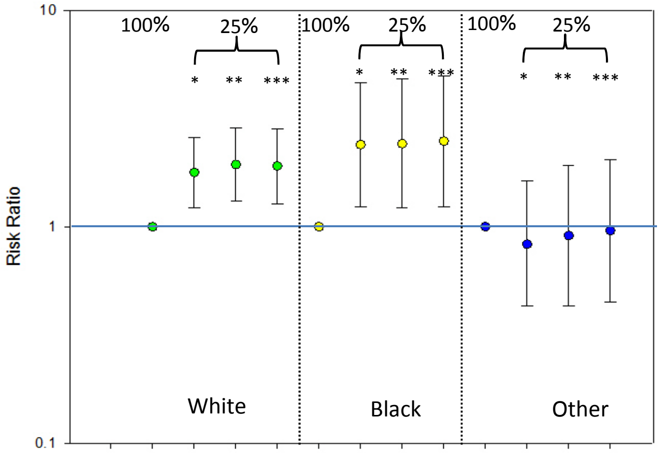

Supplement: Additional file 1: Figure S1. — The effect of dialysis facility opt-in status on outcomes, by race. A. Monthly EPO dose. B. Hb concentration. C. Likelihood of having Hb <10 g/dL. D. Likelihood of having Hb >=12 g/dL. *Unadjusted; **Adjusted for insurance (EPO dose, Hb level, percentage of subjects with Hb >=12 g/dL); ***Adjusted for insurance, age, sex, and time on dialysis. EPO, epoetin alfa; Hb, hemoglobin. Bars represent 95% confidence intervals. [file 12882_2015_59_MOESM1_ESM.pdf]
